# Impact of the diverse cardiotonic steroids on beta-amyloid precursor protein level

**DOI:** 10.3389/fphar.2025.1723629

**Published:** 2025-12-11

**Authors:** Irina Yu. Petrushanko, Denis R. Lisitskii, Maria A. Strelkova, Anna P. Tolstova, Artemy P. Fedulov, Filipp A. Filonov, Vladimir A. Mitkevich, Alexander A. Makarov

**Affiliations:** Engelhardt Institute of Molecular Biology, Russian Academy of Sciences, Moscow, Russia

**Keywords:** beta amyloid, APP, Src kinase, cardiotonic steroids, Na,K-ATPase

## Abstract

Endogenous cardiotonic steroids (CTS), which are specific ligands of Na,K-ATPase, have been detected not only in blood plasma but also in cerebrospinal fluid and in the brain tissue. Consequently, the role of CTS in the central nervous system has gained increasing relevance. Na,K-ATPase serves not only as a receptor for CTS but also as a target for beta-amyloid (Aβ_42_). Previously, we demonstrated that ouabain binding to Na,K-ATPase prevents Aβ_42_-induced activation of Src kinase and the subsequent change in the level of the amyloid precursor protein (APP) in human neuroblastoma SH-SY5Y cells. In this study, we characterized the effects of other CTS—marinobufagenin, bufalin, and digoxin, on APP level in these cells. We found that, unlike ouabain, bufalin and digoxin increased APP levels in cells independently of Aβ_42_. Marinobufagenin amplified the increase of the general APP level caused by Aβ_42_. In contrast, the addition of Aβ_42_ in the presence of bufalin or digoxin does not further elevate APP levels. Src kinase activation is observed only with marinobufagenin. This suggests that unlike Aβ_42_, which activates Src kinase, the CTS-induced rise in APP occurred through a Src-independent pathway. CTS does not lead to the accumulation of APP in neurites. Furthermore, ouabain, marinobufagenin, and digoxin reduce the rise in APP that Aβ_42_ induces in neurites. Molecular modeling data indicate that CTS binding to Na,K-ATPase alters the number of contacts formed between the enzyme and subsequently bound Aβ_42_. It means that CTS binding to Na,K-ATPase alters its interaction with Aβ_42_. Taken together, these results show that endogenous CTS are important regulators that can maintain the balance between APP and beta-amyloid in the brain.

## Introduction

Cardiotonic steroids (CTS) are specific ligands of Na,K-ATPase. At high (micromolar and millimolar) concentrations CTS inhibit the enzyme, while at lower concentrations these ligands activate a number of signaling cascades (Xie and Askari, 2002; Pierre and Xie, 2006; Aperia, 2007; Orlov et al., 2021). CTS are classified into two groups: cardenolides, characterized by a five-membered lactone ring (for example, ouabain and digoxin), and bufadienolides, which possess a six-membered lactone ring (bufalin and marinobufagenin). Additionally, CTS may contain a sugar moiety, as in ouabain and digoxin, or lack it, as in bufalin and marinobufagenin. Both CTS types have been detected in human blood and urine (Schneider et al., 1998; Kawamura et al., 1999; Goto et al., 1990; Lichtstein et al., 1993; Yoshika et al., 1979; Bagrov et al., 1979; Fedorova et al., 1993; Haddy and Overbeck, 1976; Hamlyn et al., 1991; Komiyama et al., 2005; Orlov et al., 2021).

Endogenous CTS are present in the blood of healthy donors at subnanomolar concentrations, with levels increasing to nanomolar concentrations in various pathological conditions. Initially, it was demonstrated that CTS synthesis occurs in the adrenal glands (Hamlyn et al., 1991). However, current evidence indicates that CTS are also produced in brain tissue (Kawamura et al., 1999; Gomez-Sanchez et al., 2010; Taves et al., 2011; Hamlyn et al., 2014; Takahashi et al., 2008; Leenen et al., 2017). The concentration of CTS in cerebrospinal fluid (CSF) exceeds that in blood by several folds; notably, ouabain levels in CSF are four times higher than in serum (Dvela et al., 2012). While ouabain does not cross the blood-brain barrier (Abaimov et al., 2024), the more hydrophobic digoxin enters the brain from the bloodstream (Taskar et al., 2017), exerting various effects within the central nervous system (Mallakh et al., 1995). Due to its independent synthesis in the brain, the level of CTS remains stable in the hypothalamus even when blood concentrations decrease following adrenal gland removal in animals (Weidemann et al., 2004; Manunta et al., 2006). Currently, increasing evidence is emerging regarding the functions of CTS in the brain (Leenen et al., 2017; Hodes et al., 2018; Blaustein and Hamlyn, 2020; Castro de Jesus et al., 2024).

Disruption of endogenous CTS synthesis involving α2-and α3-isoforms of Na,K-ATPase impairs learning and memory, affecting mood and behavior in animals (Blaustein and Hamlyn, 2020). The levels of endogenous CTS in blood are altered in neurodegenerative diseases such as Alzheimer’s disease (AD) and Parkinson’s disease (Orellana et al., 2016; Lopachev et al., 2018; Fedorova et al., 2020). Particularly, in the APP/PS1 mouse model of AD, a reduction in marinobufagenin in serum was observed (Fedorova et al., 2020). Restoring its level through exogenous marinobufagenin administration led to decreased IL-6 in the brain and reduced neuroinflammation (Fedorova et al., 2020). Although current data do not show changes in brain CTS levels in AD, it has been demonstrated that the production of sex hormones, also synthesized in the hypothalamus, is reduced in patients at early stages of AD (Laurell et al., 2025). Similar hypothalamic dysfunction has been observed in a murine model of AD (APP/PS1) without any interventions (Qi et al., 2024).

In all, it can be hypothesized that alterations in CTS levels contribute to neurodegeneration in AD. In its turn, the beta-amyloid (Aβ) plays a crucial role in AD pathogenesis (Lane et al., 2018). It is formed from amyloid precursor protein (APP) through the action of gamma- and beta-secretases (Orobets and Karamyshev, 2023), which colocalization is necessary for Aβ production (Sun and Roy, 2018). From the Golgi network, APP is transported to the plasma membrane, where it is cleaved via a non-amyloidogenic pathway, resulting in soluble APP formation (Müller et al., 2017), and an amyloidogenic pathway, leading to the formation of Aβ. In neuronal cells, APP is expressed on the surface of somas, axons, dendrites, and synaptic sites (Sun and Roy, 2018). With aging, APP processing intensifies. Besides, APP tends to accumulate in neurites of aging neurons (Burrinha et al., 2021; Burrinha and Guimas Almeida, 2022).

Na,K-ATPase functions not only as a receptor for CTS but also for Aβ_42_, with binding to Aβ_42_ leading to activation of Src kinase and inhibiting the enzyme at high doses (Petrushanko et al., 2016; Petrushanko et al., 2022). Previously, we demonstrated that Aβ_42_ induces a Src-dependent increase in APP levels in human neuroblastoma cells, an effect that is completely prevented by ouabain, which itself does not affect Src kinase activation following a 30-min incubation. However, it inhibits the Aβ_42_-induced elevation of APP levels by blocking the Aβ_42_-induced activation of Src kinase (Petrushanko et al., 2025). Different CTS bind Na,K-ATPase in distinct ways, inducing conformational changes of the enzyme (Klimanova et al., 2015) that may alter their interactions with other ligands. Alongside ouabain, some of the most prevalent and extensively studied endogenous CTS in humans are marinobufagenin, bufalin, and digoxin (Bagrov et al., 1979; Fedorova et al., 1993; Lichtstein et al., 1993; Schneider et al., 1998; Kawamura et al., 1999; Weinberg et al., 1992; Goto et al., 1990; Orlov et al., 2021). Digoxin is also a widely used pharmaceutical agent in the treatment of cardiovascular diseases (Patocka et al., 2020).

In this study, the effects of marinobufagenin, bufalin, and digoxin on APP production and Src kinase activation were evaluated in neuroblastoma cells, both in the absence and presence of Aβ.

## Materials and methods

### Cell line

Human neuroblastoma cell line SH-SY5Y obtained from the American Type Culture Collection was cultured in RPMI-1640 medium (Gibco, ThermoFisher Scientific, Waltham, MA, United States), containing 10% fetal bovine serum (FBS; Gibco, ThermoFisher Scientific, MA, United S), 100 units/mL penicillin, 100 μg/mL streptomycin and GlutaMax (Gibco, ThermoFisher Scientific, Waltham, MA, United States). Culture maintenance was performed in cultural flasks T-25 and T-75 at 37 °C in humid atmosphere, containing 5% CO_2_. SH-SY5Y cells were dissociated via washing with Versene solution (Gibco, ThermoFisher Scientific, Waltham, MA, United States) and 0.05% trypsin-EDTA (Gibco, ThermoFisher Scientific, Waltham, MA, United States) digestion at 37 °C during 5 min. Passages did not exceed 15. For confocal microscopy cells were seeded on 35 mm glass-based Petri dishes (Nunc, Rochester, NY, United States, 150680) in a quantity 15000 per dish. For Western blotting, redox parameters and Ca^2+^ level measurements SH-SY5Y cells were grown on 6- and 12-well plates until 80%–90% confluency was achieved.

### Aβ_42_ preparation

Synthetic peptide Aβ_42_: [H2N]-DAEFRHDSGYEVHHQKLVFFAEDVGSNKGAIIGLMVGGVVIA-[COOH] was obtained from Biopeptide (San Diego, CA, United States). Preparation of the monomeric form of Aβ_42_ was performed as described elsewhere. Cold hexafluoroisopropanol (Fluka) was added to dry Aβ_42_ until peptide concentration 1 mM was achieved. After 1-h incubation peptide solution was transferred on ice for 10 min and aliquoted into microcentrifuge tubes (0.56 mg Aβ_42_ per tube). Aliquots were dried under vacuum using Eppendorf Concentrator 5301. Dried peptide films were stored at −80 °C. 2.5 mM stock solution was prepared by dissolving 0.22 mg of dry peptide in 20 μL of 100% anhydrous DMSO (Sigma-Aldrich, St. Louis, MO, United States) and 1-h incubation. The required concentration of Aβ_42_ was achieved by dilution of stock solution with RPMI-1640 medium or Tyrode solution. An equivalent volume of pure DMSO was added to control probes. Only fresh-dissolved Aβ_42_ was used in experiments.

### Treatment of cells with cardiotonic steroids and Aβ_42_

Incubation of SH-SY5Y cells on plates or dishes with amyloid peptide required medium replacement with FBS-free RPMI-1640. For the flow cytometry studies SH-SY5Y cells were dissociated and suspended in Tyrode solution with following staining before incubations with CTS and Aβ_42_. Aβ_42_ stock solution was diluted and added to samples in final concentration 100 nM. Beta-amyloid effects were studied by SH-SY5Y incubation for 30 min. Influence of cardiotonic steroids was estimated by pre-treatment for 30 min before adding of Aβ_42_ solution. Digoxin (ThermoFisher Scientific), ouabain (Fluka), marinobufagenin (Cayman) and bufalin (Sigma Aldrich) were used in concentration 100 nM. All incubations were performed at 37 °C in humid atmosphere, containing 5% CO_2_.

### Estimation of APP level and Src kinase activation

Cells were grown on 6- and 12-well plates until 80%–90% confluency was achieved. Medium was replaced with FBS-free RPMI-1640 and cells were incubated with Aβ_42_, cardiotonic steroids and Src kinase inhibitor 1. After incubation wells were washed with Ca^2+^/Mg^2+^ PBS and cells were lysed with RIPA buffer (ThermoFisher Scientific, Waltham, MA, United States, 89900), containing protease inhibitors cocktail (Roche, 11836145001), phosphatase inhibitors cocktail (Roche, 4906837001), PMSF (Roche, 10837091001) and 5 μM thiorphan (Cayman Chemical, Ann Arbor, MI, United States, 15600), with stirring for an hour at 4 °C. Lysates were centrifuged at 4 °C and 16000 g for 10 min and supernatants were collected.

The cell lysates were separated via 10% SDS-PAGE electrophoresis and transferred to a PVDF-membrane (Bio-Rad, Hercules, CA, United States, 1620137). Membranes were blocked in 5% nonfat milk in TBST (50 mM Tis-HCl, pH 7.4, 150 mM NaCl, 0.1% Tween-20) for an hour and were incubated with monoclonal primary rabbit antibodies specific to APP (dilution in 5% milk-TBST 1/5000, Abcam Limited, Discovery Drive, Cambridge Biomedical Campus, Cambridge, United Kingdom, ab32136), Src-kinase (dilution in 5% milk-TBST 1/1000, Cell Signaling Technology, Danvers, MA, United States, 2108S) and Phospho-Src Family (Tyr416) (Cell Signaling Technology, Danvers, MA, United States, 6943S) overnight at 4 °C. Then, the membranes were washed in TBST and incubated with secondary antibodies, conjugated with HRP (dilution in TBST 1/5000, Hytest, Moscow, Russia, GARC). Imaging of the membranes was performed using SuperSignal™ West Femto Maximum Sensitivity Substrate kit (ThermoFisher Scientific, MA, United States, 34096) and Bio-Rad ChemiDoc MP instrument (Bio-Rad, Hercules, CA, United States). Densitometric analysis was performed with Image Lab 6.0.1 program (Bio-Rad, Hercules, CA, United States). Results were expressed as APP levels, normalized on that in control (APP, %), or phospho-Src/Src ratio (p-Src/Src, fold change).

### Amyloid precursor protein distribution studies

SH-SY5Y cells were cultured on Petri dishes until 50% confluency was achieved. Medium was replaced with FBS-free RPMI-1640 and cells were preincubated with CTS (ouabain, digoxin, bufalin and marinobufagenin) during 30 min and then with 100 nM amyloid during 120 min. For each time point own control without addition of Aβ_42_ was performed. As incubations ended cells were washed with ice-cold Ca^2+^/Mg^2+^ PBS and fixed in 4% para-formaldehyde solution for 10 min. At the end of fixation cells were washed with Ca^2+^/Mg^2+^ PBS and treated with monoclonal rabbit antibodies against N-terminal extracellular domain of amyloid precursor protein (dilution in PBS 1/100, Abcam Limited, Discovery Drive, Cambridge Biomedical Campus, Cambridge, United Kingdom, ab126732, at 4 °C overnight. Next day, samples were washed with Ca^2+^/Mg^2+^ PBS and incubated for 2 h at room temperature with secondary antibodies, conjugated with AlexaFluor® 488 (Ex/Em = 495/519 nm, dilution in PBS 1/500, Abcam Limited, Discovery Drive, Cambridge Biomedical Campus, Cambridge, United Kingdom, ab150077). Before the imaging nuclei were stained with NucBlue (Ex/Em = 360/460, Invitrogen, ThermoFisher Scientific, MA, United States, R37605).

### Assessment of the redox status of the cells

SH-SY5Y cells were grown on 12-well plates at 37 °C in humid atmosphere, containing 5% CO_2_, and suspended in Tyrode solution after achieving 80%–90% confluency. Then, suspensions from every well were divided into two parts, every sample was stained simultaneously with GSH- and ROS- specific dyes. The ROS level was assessed using the dihydrorodamine 123 (DHR) dye (Ex/Em = 488/525 nm; Invitrogen, ThermoFisher Scientific, MA, United States, D23806, used in a concentration 5 μM). The monobromobimane dye (Ex/Em = 393/490 nm, Sigma-Aldrich, St. Louis, MO, B4380, used in a concentration 20 μM) was used for the reduced glutathione (GSH) staining. All parameters were recorded for the cells with intact membrane. The dyes were incubated at 37 °C for 30 min. Detection of the cells with damaged membrane (dead cells) was performed by staining with propidium iodide (PI) (Ex/Em = 535/617 nm, Sigma-Aldrich, St. Louis, MO, P4170, used in a concentration 10 μg/mL) 1 minute before measurement. When assessing the levels of ROS, GSH, dead cells were excluded from consideration. The cells were analyzed using a flow cytometer BD LSR Fortessa (Becton Dickinson, Franklin Lakes, NJ, United States).

### Molecular modeling

The previously constructed human Na,K-ATPase model was embedded into DDPC bilayer membrane and used as a starting structure (Adzhubei et al., 2022). Na,K-ATPase:CTS complexes were modeled using docking in the Autodock Vina software (Hassan et al., 2017). The resulting complexes were then simulated using conventional molecular dynamics (MD) for 100 ns in the GROMACS software environment (Abraham et al., 2015) to obtain equilibrium conformations, which were extracted from the MD trajectories by clustering procedure. Force field topologies and optimized PDB structures of CTS were generated by ATB web server (Stroet et al., 2018). The equilibrium model structure of Aβ_42_ was used for docking (Tolstova et al., 2024). Servers for protein docking do not handle non-protein ligands and small molecules. Therefore, CTS were not taken into account during docking of Aβ_42_ to CTS:Na,K-ATPase complexes. Targeted docking of the Aβ isoforms to the equilibrium structures of the Na,K-ATPase extracted from CTS:Na,K-ATPase complexes was performed on the HADDOCK (Zundert et al., 2016), GRAMM (Tovchigrechko and Vakser, 2006), and PatchDock (Schneidman-Duhovny et al., 2005) web servers. Docking was performed to the previously determined interaction interface with Aβ including residues 119-AATEE-123, 310-SLILEY-315, 887-RVDWDDR-893 in the α-subunit, and residues 84-QKTEI-88 in the β-subunit (Adzhubei et al., 2022). The resulting complexes from the docking were analyzed using QASDOM server (Anashkina et al., 2018). Data were summarized for all representative conformations of each Aβ isoform and averaged over the conformational ensemble of each isoform. The resulting complexes of CTS:Na,K-ATPase and the best ranking docking poses of Aβ_42_ to that complexes are available on Zenodo (https://doi.org/10.5281/zenodo.17334488).

### Statistical analysis

All experimental data are shown as mean values ± standard deviations of mean (SD) or standard error of mean (SEM), with the number of independent experiments indicated in Figure legends. The data sets were tested for normal distribution using the Shapiro-Wilk test. For a data set where the data in all groups have a normal distribution, statistical difference between experimental two groups was analyzed by unpaired t-test, differences between three or more groups were estimated by one-way ANOVA with *post hoc* Tukey or Bonferroni test for multiple comparisons. Probability values (p) less than 0.05 were considered significant. For non-normal data set, Kruskal–Wallis test with *post hoc* Dunn’s multiple comparisons test were used. Statistical analysis was performed using GraphPad Prism 9.1.2 software (GraphPad Software Inc., San Diego, CA, United States).

## Results

### Cardiotonic steroids exert distinct impact on Aβ_42_-induced APP growth

**FIGURE 1:**
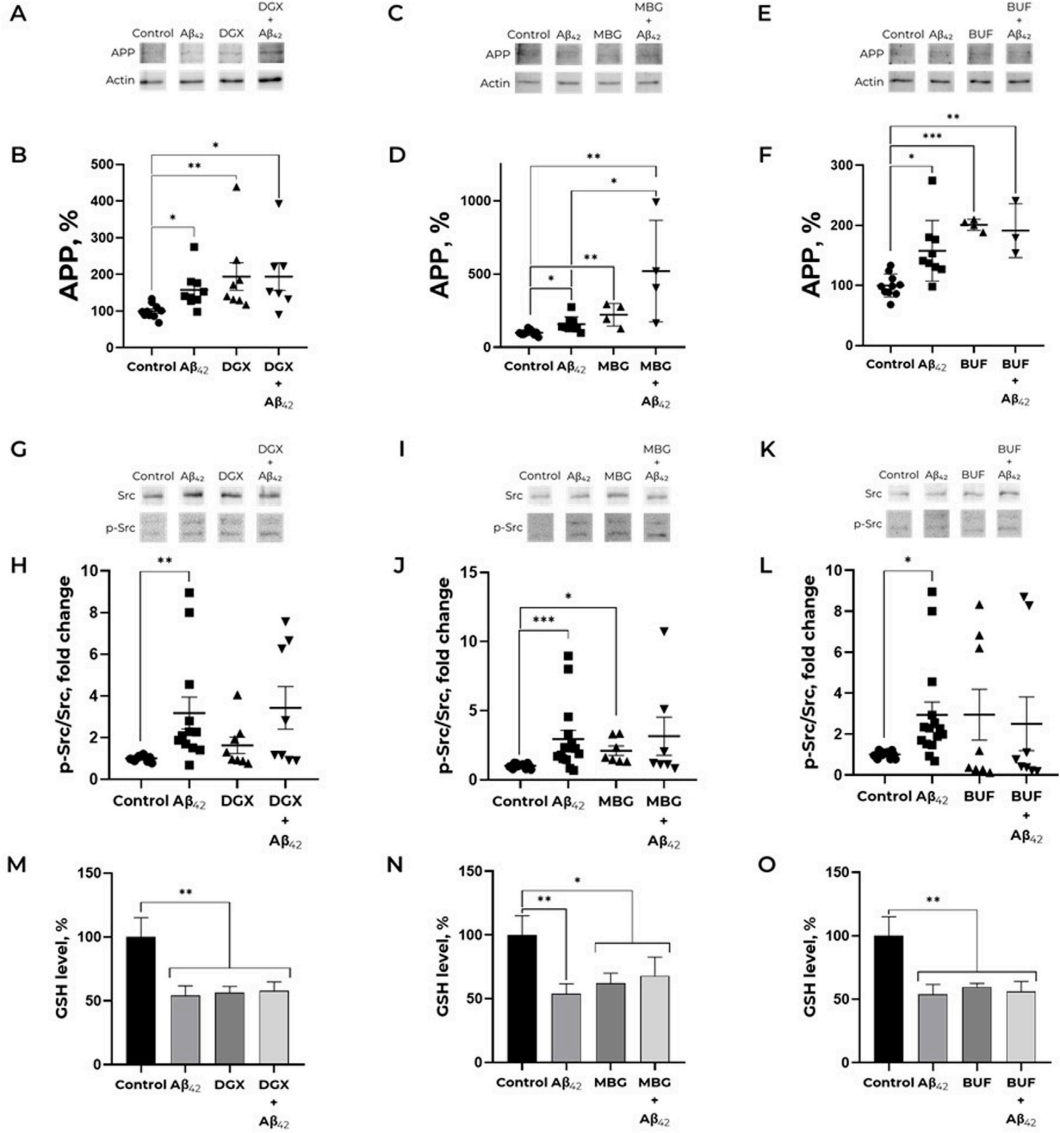
Effect of cardiotonic steroids and Aβ_42_ on the APP level **(A–F)**, Src kinase activation **(G-L)** and reduced glutathione level **(M-O)**. **(A,B)**. Dependence of APP expression on the presence of Aβ_42_ and/or digoxin. Kruskal–Wallis test with *post hoc* Dunn’s test for multiple comparisons were used. **(C,D)** Impact of marinobufagenin and Aβ_42_ on the APP level. One-way ANOVA with *post hoc* Bonferroni test for multiple comparisons were used. **(E,F)** Impact of Aβ_42_, bufalin and Aβ_42_ after bufalin pre-treatment on APP expression. One-way ANOVA with *post hoc* Bonferroni test for multiple comparisons were used. The ratio APP to actin has been calculated for every single membrane. **(G,H)** Dependence of Src kinase activation on the presence of Aβ_42_ and/or digoxin. **(I,J)** Impact of marinobufagenin and Aβ_42_ on Src activation. **(K, L)** The ratio of p-Src to total Src under bufalin and/or Aβ_42_ treatment. p-Src/Src is equal to the ratio of phospho-Y419 Src to the total Src kinase signals. Kruskal–Wallis test with *post hoc* Dunn’s test for multiple comparisons were used. The APP levels and phosphorylated and total Src levels were measured with Western blot in SH-SY5Y human neuroblastoma cells treated with 100 nM Aβ_42_, 100 nM CTS or both for 30 min and normalized for control. **(M)** Reduced glutathione level after treatment with Aβ_42_, digoxin and their combination. **(N)** An impact of Aβ_42_ and marinobufagenin on intracellular GSH. **(C)** GSH level under amyloid and marinobufagenin treatment. **(O)** Alterations in the reduced glutathione levels under exposure with Aβ_42_ and bufalin. The SH-SY5Y human neuroblastoma cells were harvested stained with monobromobimane for GSH measurements and incubated with 100 nM Aβ_42_ and 100 nM CTS for 30 min. Mean values ±SEM (B, D, F, H, J, L) or SD (M, N, O) from at least three independent experiments are shown. * – p < 0.05, ** – p < 0.01, *** – p < 0.001 compared to the control.

To evaluate the effect of marinobufagenin, bufalin, and digoxin on APP level in human neuroblastoma cells we used the 100 nM concentration, which was previously used for ouabain (Petrushanko et al., 2025). These CTS do not induce cell death in SH-SY5Y neuroblastoma cells after 24-h exposure at concentrations up to 100 nM (Supplementary Figure S1). Treatment of SH-SY5Y cells with 100 nM digoxin resulted in a significant increase in APP level compared to the control (Figures 1A,B). Subsequent addition of 100 nM Aβ_42_ did not produce any further increase in APP levels (Figures 1A,B). In the presence of Aβ_42_ and marinobufagenin combined, the effect of APP accumulation was the most pronounced, compared to the separate effect of Aβ_42_ (Figures 1C,D). This indicates that marinobufagenin potentiates the Aβ_42_-induced increase in APP levels. Treatment with bufalin or digoxin elevated APP level to a degree comparable to that of Aβ_42_ (Figures 1A,B,E,F). The joint application of bufalin and Aβ_42_ resulted in APP levels similar to those observed with each compound administered separately (Figures 1E,F). (Full-size Western blot membranes are shown in the Supplementary Figure S2A-D, S3A,B, S4A,B).

**FIGURE 2:**
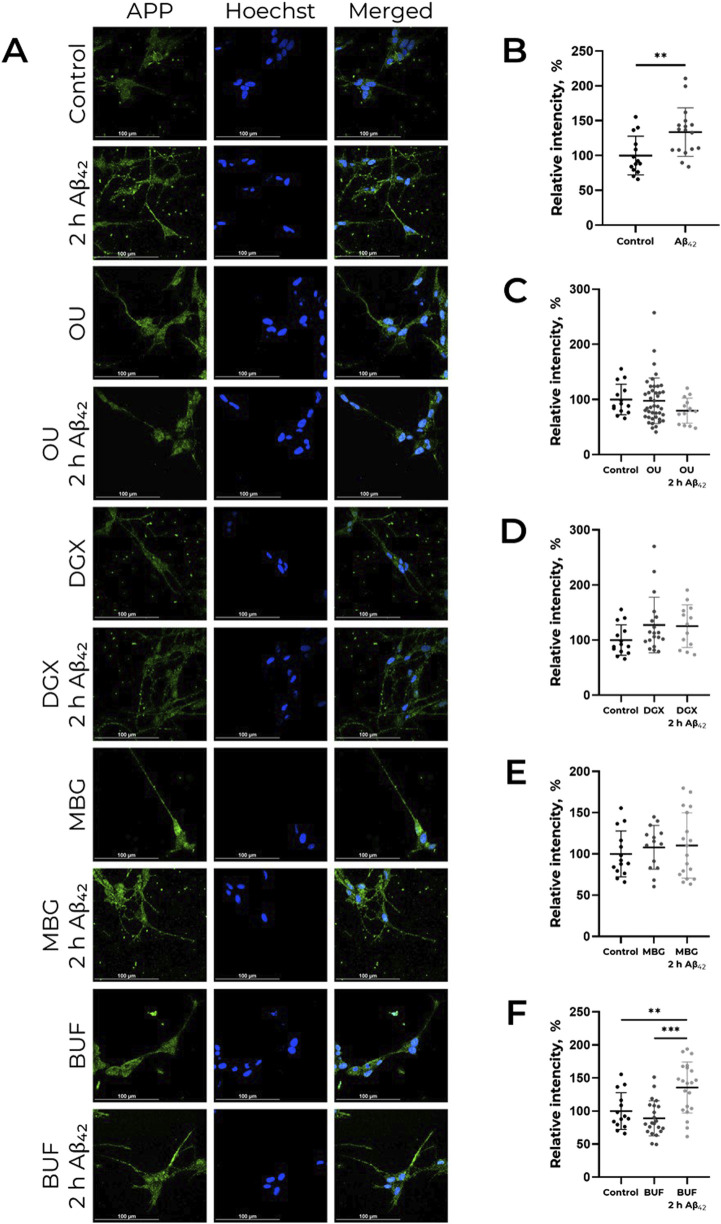
CTS impact on Аβ_42_-induced APP increase in neurites in human neuroblastoma cells SH-SY5Y. **(А)** Localization of APP in cells in presence of CTS or CTS with Аβ_42_ combined, incubation time 2 h. The data is provided on the APP distribution change under the action of Аβ_42_ **(B)**, ouabain (OU), **(C)** digoxin (DGX), **(D)** marinobufagenin (MBG), **(E)** and bufalin (BUF), **(F)** All compounds were used in 100 nM concentration. APP was labeled with AlexaFluor® 488-conjugated antibodies and is indicated by green color, nuclei are stained with NucBlue (Hoechst 33342) and indicated by blue color. For each incubation time, the ratio of fluorescence in neurites and cell bodies was calculated, and the values were normalized to the control. One-way ANOVA with *post hoc* Tukey test for multiple comparisons were used. Mean values ± SD, n = 3 from at least three independent fields are shown. ** – p < 0.01, *** – p < 0.001 compared to the control.

Previously we found that Aβ_42_ does not only cause APP increase, but elevates its content in neurites, which is observed already after 2 h of incubation (Petrushanko et al., 2025). We evaluated the effect of CTS, namely, ouabain, digoxin, bufalin, and marinobufagenin, on APP distribution in cells. It was revealed that none of the investigated CTS alter the APP content in neurites (Figure 2). At the same time, ouabain, digoxin, and marinobufagenin prevent the APP increase in neurites under the action of Aβ_42_, in contrast to bufalin, which does not affect Aβ_42_-induced alterations in APP distribution (Figure 2).

### Cardiotonic steroids affect Aβ_42_-induced Src kinase activation

The Aβ_42_-induced increase in APP levels is mediated by Src kinase activation (Petrushanko et al., 2025). However, digoxin and bufalin increased APP levels without inducing Src kinase activation over the investigated time period (Figures 1G,H,K,L). Pre-incubation with digoxin or bufalin prior to the addition of Aβ_42_ abolished the Aβ_42_-induced activation of Src kinase. In contrast, marinobufagenin was the only cardiotonic steroid found to activate Src kinase within 30 min of incubation (Figures 1I,J). Nevertheless, the subsequent addition of Aβ_42_ did not lead to a further increase in Src activation, a result similar to that observed with digoxin and bufalin. Thus, studied CTS, with the exception of marinobufagenin, do not activate Src kinase but further prevent Aβ_42_-induced Src activation. (Full-size Western blot membranes are shown in the Supplementary Figure S2E-H, S3C-F, S4C-F).

### СTS effect on Aβ_42_-induced decrease in GSH level

To find out whether CTS effect on activation of Src kinase, which is a redox-sensitive protein (Heppner et al., 2018; Yang et al., 2020), is mediated by altered redox status of cells, the levels of GSH in SH-SY5Y cells exposed to 100 nM CTS before the incubation with 100 nM Aβ_42_ were evaluated (Figures 1M–O). The incubation of SH-SY5Y cells with all examined CTS resulted in the same decrease in GSH levels as incubation with Aβ_42_. At the same time, in cells exposed to a concurrent treatment with CTS and Aβ_42_, the GSH level was not altered compared to those with Aβ_42_ or CTS alone (Figures 1M–O). Of note, СTS do not alter the effect of Aβ_42_ on ROS levels (Supplementary Figure S5).

### MD modeling of Na,K-ATPase conformations in complexes with CTS

**FIGURE 3:**
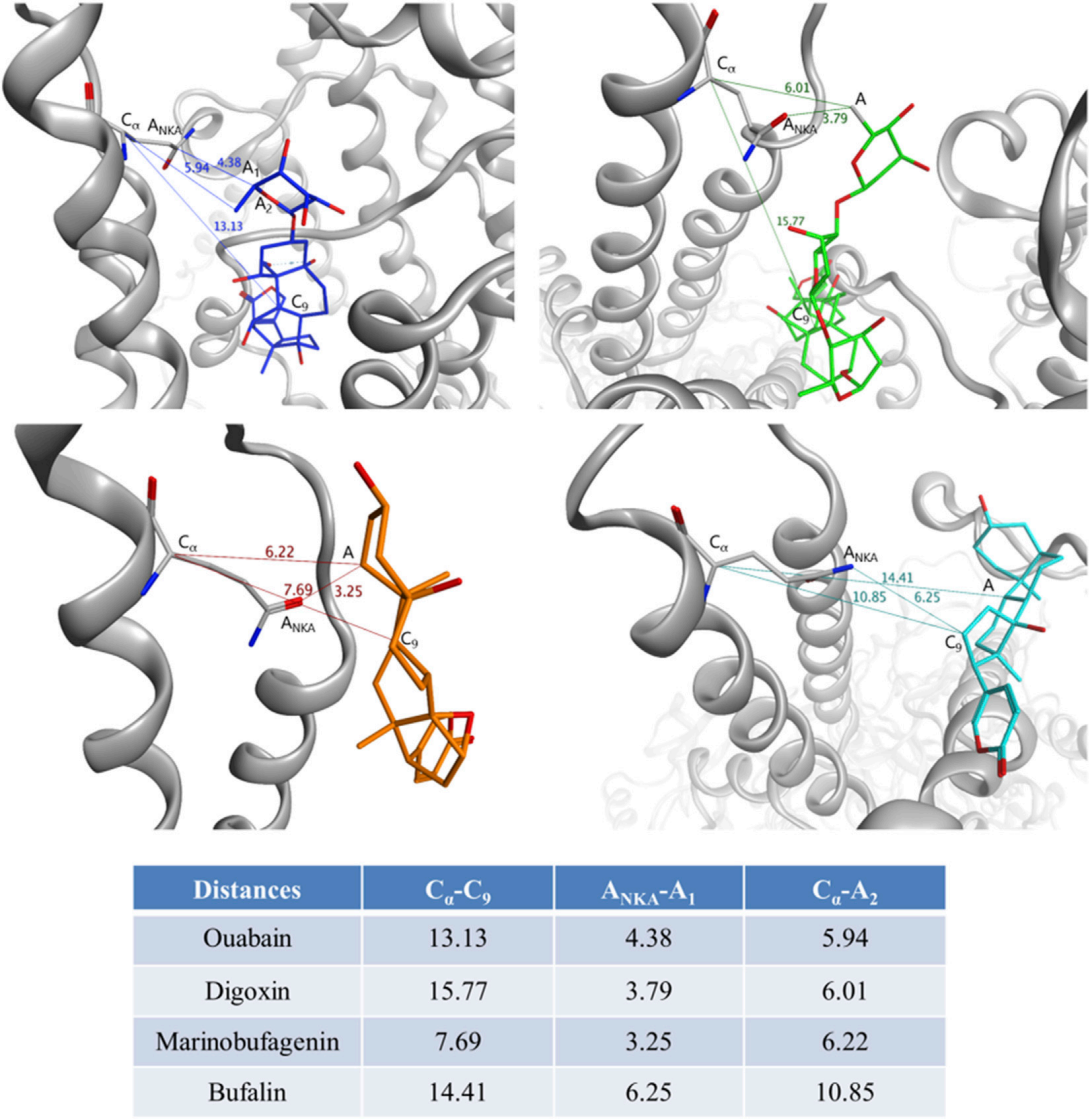
Distances between CTS and Gln118 residue of Na,K-ATPase measured for the representative complexes obtained by 100 ns CMD. For Gln118 of Na,K-ATPase, C_α_ is C_α_ atom, A_NKA_ is the heavy atom nearest to CTS. For each CTS, C_9_ is the C_9_ atom of the steroid core, A_1_ is the heavy atom of CTS nearest to Gln118, A_2_ is the heavy atom of CTS nearest to C_9_, A is the atom consistent to A_1_ and A_2_. Na,K-ATPase is shown in grey, ouabain is shown in blue, digoxin is shown in green, marinobufagenin is shown in orange, bufalin is shown in cyan.

Equilibrium conformations of Na,K-ATPase complexed with various CTS (Figure 3; Supplementary Figure S6) were obtained. Using 100 ns of conventional MD (CMD) simulations, we performed clustering analysis. From the resulting trajectory, a representative structure was extracted from the most populated cluster. This structure represents the equilibrated conformation, which was subsequently utilized in docking simulations. This approach ensures that the CTS are docked to a stable and biologically relevant human Na,K-ATPase structure. The docking results with the highest scores were further relaxed with CMD simulation. The resulting complexes of CTS:Na,K-ATPase can be found on Zenodo (https://doi.org/10.5281/zenodo.17334488).

To analyze the variability of the Na,K-ATPase conformations in complexes with CTS, we performed a structural alignment and built RMSD matrix between four resulting MD Na,K-ATPase:CTS complexes together with available X-ray structures 4HYT (ouabain Na,K-ATPase), 4RES (bufalin:Na,K-ATPase), 4RET (digoxin:Na,K-ATPase), 7DDH (digoxin:Na,K-ATPase) and 7DDL (bufalin:Na,K-ATPase) (Supplementary Figure S7). RMSD matrix showed significant structural differences, especially in intracellular structured domains, between the Na,K-ATPase conformations bound to different CTS (Supplementary Figures S6, S7). Molecular modeling revealed that all conformations of human Na,K-ATPase in complex with CTS were more similar to each other than to the X-ray data for Na,K-ATPase structures obtained for other organisms (Supplementary Figure S7). Within this set, however, the ouabain:Na,K-ATPase complex was an outlier, showing the greatest differences in terms of RMSD (Supplementary Figure S7).

### CTS orientation in the Na,K-ATPase entry

To investigate the structural impact of different CTS on the extracellular entry part of the Na,K-ATPase, we performed a targeted structural alignment and built RMSD matrix between all Na,K-ATPase structures. This alignment focused on two key regions: the area immediately preceding the ion channel, where our data (Adzhubei et al., 2022) suggest Aβ interact, and the channel itself, which is the CTS binding site (Supplementary Figure S7A,B). The RMSD matrices revealed that the conformational differences among Na,K-ATPase structures in complex with various CTS were less pronounced within this specific, functionally critical region compared to the overall protein structure. Nonetheless, some distinctions in mutual arrangement of M1-M2 and M3-M4 helices (Supplementary Figures S6,S7) were evident and can be crucial for CTS binding. The ouabain:Na,K-ATPase complex consistently showed the greatest structural deviation from the other CTS-bound complexes. In contrast, the digoxin:Na,K-ATPase complex exhibited the highest degree of structural similarity to the complexes formed with all other CTS.

According to our modeling, CTS generally occupied the same region within the channel and remained relatively immobile, with the exception of digoxin (Figure 3; Supplementary Figure S6). Digoxin is larger and more flexible than the other CTS and therefore its non-steroid part was flexible during MD. However, its steroid moiety remained mostly immobile. To estimate the difference in CTS position and orientation, we have measured several distances between CTS atoms and residue Gln118 of Na,K-ATPase α-subunit (Figure 3). This residue can mark the Na,K-ATPase channel entry point as it is located on the loop between M1 and M2 α-helices close to the membrane surface. It is also known as one of the residues participating in the Na,K-ATPase:CTS interaction (Tverskoi et al., 2021). The distances between C_α_ atom of Na,K-ATPase Gln118 residue and C_9_ atom of CTS steroid core representing its center for various CTS are shown in Figure 3. Since all the CTS are different in size, the distances between C_α_ or nearest heavy atoms of Na,K-ATPase and CTS were also measured as an indicator of depth of CTS immersion in the channel (Figure 3). According to these data, bufalin was positioned deeper within the channel than the other CTS, while marinobufagenin was located most superficially.

### Impact of CTS binding on the Aβ_42_:Na,K-ATPase interacting interface

**FIGURE 4:**
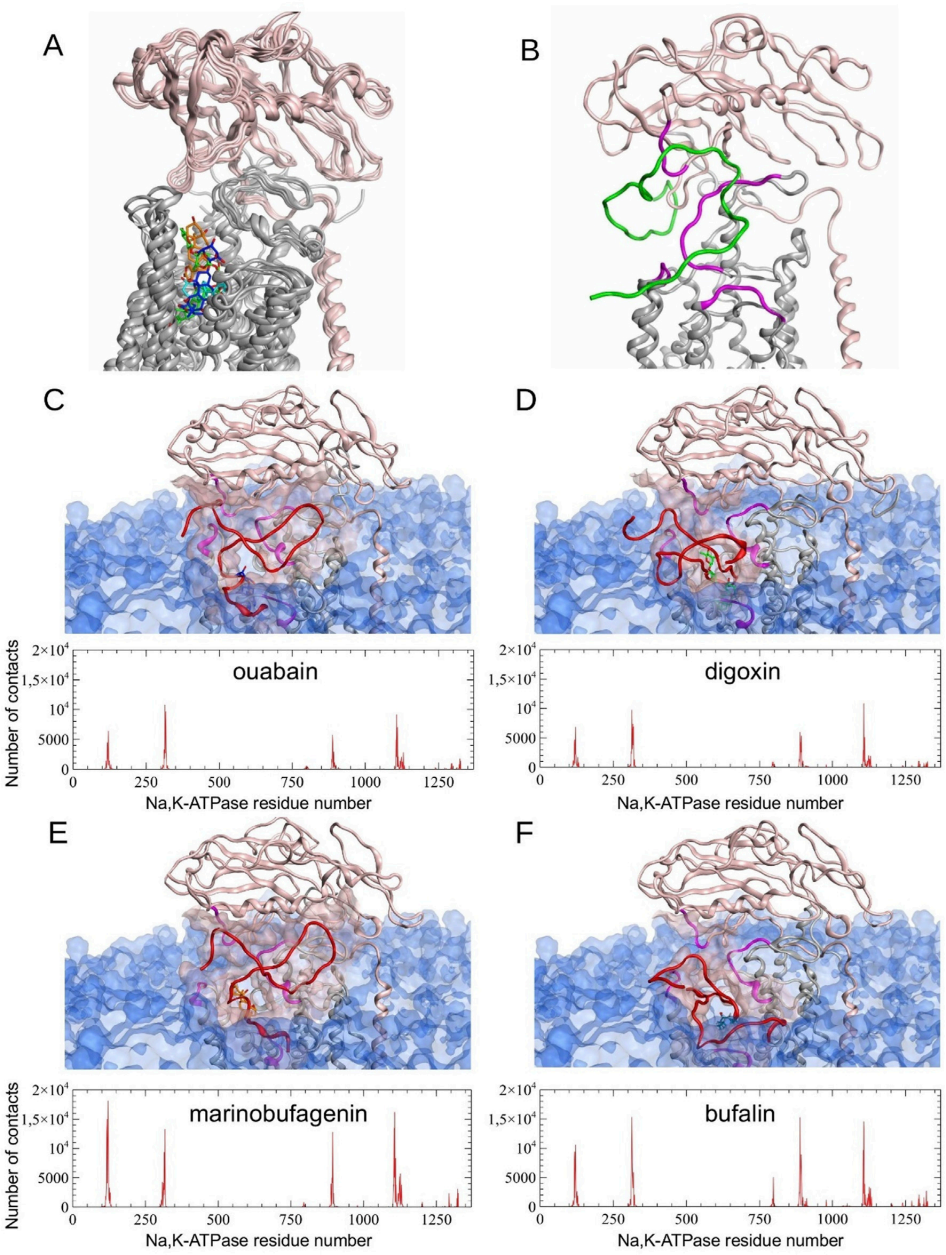
Molecular modeling results. **(A)** Structural alignment of four different Na,K-ATPase:CTS complexes relaxed by 100 ns CMD simulation. **(B)** Representative E2P Na,K-ATPase:Aβ_42_ complex without CTS obtained by 100 ns CMD modeling (Adzhubei et al., 2022) **(C–F)** Results of Aβ_42_ peptide docking to Na,K-ATPase in a complex with CTS. Histograms of contacts (total number of contacts for each receptor residue, calculated across all docking complexes) were calculated for complexes obtained using GRAMM, PatchDock and HADDOCK docking servers. The best scored representative complexes selected by QASDOM server: **(C)** Na,K-ATPase:ouabain, **(D)** Na,K-ATPase:digoxin, **(E)** - Na,K-ATPase:marinobufagenin, **(F)** - Na,K-ATPase:bufalin. In the contact histograms, residues 1–1023 belong to α-subunit, residues 1024–1327 belong to β-subunit, and residues 1328–1392 belong to γ-subunit. In the images of the representative complexes, the interaction interface on Na,K-ATPase is highlighted in magenta and transparent pink, and CTS in blue for ouabain, green for digoxin, orange for marinobufagenin and cyan for bufalin. Membrane surface is highlighted with transparent blue. α-subunit of Na,K-ATPase is colored with gray and β-subunit is colored with pink. Docked Aβ_42_ is colored with red. Modeled Aβ_42_ in **(B)** is colored with green.

The differences between MD-derived Na,K-ATPase conformations bound to different CTS (Supplementary Figures S6,S7) affect the consequent Aβ_42_ binding. We observed the preservation of the previously predicted Na,K-ATPase interaction interface with Aβ (Adzhubei et al., 2022), which involves residues 119-AATEE-123, 310-SLILEY-315, and 887-RVDWDDRWIND-897 of the α-subunit and 84-QKTEI-88 of the β-subunit (Supplementary Table S1). This observation is supported by the concordance of peaks in the contact histograms of Na,K-ATPase: Aβ_42_ docking complexes (Figure 4). However, the total number of contacts varied depending on the presence of different CTS (Figure 4). Na,K-ATPase in the presence of marinobufagenin showed the highest number of contacts with Aβ_42,_ nearly double that of the complexes with digoxin or ouabain (303417 atomic contacts over 15 docking sets for marinobufagenin bound Na,K-ATPase versus 171960 and 158939 atomic contacts for digoxin and ouabain bound Na,K-ATPase, respectively). The fewest number of contacts is observed in ouabain:Na,K-ATPase complex. The binding positions of digoxin overlapped with the representative Aβ_42_ model from the docking simulations, suggesting that the digoxin itself may exert a direct influence on the Aβ_42_:Na,K-ATPase interaction. The best ranking docking poses of Aβ42 to CTS:Na,K-ATPase complexes are available on Zenodo (https://doi.org/10.5281/zenodo.17334488).

## Discussion

The diversity of cardiotonic steroids (CTS) in the human body holds profound regulatory significance. Acting as specific ligands for Na,K-ATPase, different CTS vary in their effects on cells and the signaling cascades they activate. Apart from that, their levels also change differently in numerous diseases (for rev. see Orlov et al., 2021; Pavlovic, 2020; Contreras et al., 2024). The plasma levels of the most studied endogenous CTS, ouabain and marinobufagenin, have been characterized in various pathologies, particularly cardiovascular diseases (Orlov et al., 2021). Although CTS are synthesized in the human brain (Dvela et al., 2012), data on the content and role of endogenous CTS in the brain, as well as their fluctuations in neurodegeneration, remains scarce. The diversity of CTS effects on APP level in cells, which was demonstrated in this study, allows us to consider them as direct participants in regulating Aβ levels in brain tissue and underscores the importance of their heterogeneity.

The effect we described previously, wherein Aβ_42_ increases APP levels in neuronal cells and promotes its translocation into neurites (Petrushanko et al., 2025), is mediated by Src kinase activation. This effect represents a potential mechanism for enhanced Aβ_42_ production in sporadic Alzheimer’s disease (AD) and its accumulation in neurites with age (Burrinha et al., 2021). Ouabain prevents both Src activation and the Aβ_42_-induced increase in APP levels (Petrushanko et al., 2025). However, as shown in the present study, marinobufagenin, bufalin, and digoxin exert entirely different effects on both APP and Src kinase activation (Figure 1). Digoxin and bufalin induce an increase in APP levels in cells; marinobufagenin potentiates the effect of Aβ_42_ on APP levels (Figure 1). It is noteworthy that the digoxin- and bufalin induced increase in APP is not accompanied by Src activation, indicating a mechanism distinct from that of Aβ_42_. The fact that, unlike Aβ_42_, all investigated CTS, including ouabain, do not lead to the accumulation of APP in neurites, further supports the different mechanisms of action of Aβ_42_ and CTS (Figure 2). The IC50 values for Na,K-ATPase inhibition by marinobufagenin, digoxin, and ouabain are similar (0.8, 1.2, and 2.0 μM, respectively) (Tverskoi et al., 2021). Thus, observed variety of CTS effects on the APP levels is not associated with Na,K-ATPase inhibition. We propose that the reason for these divergent effects of CTS on APP levels and Src kinase activation in cells is that binding of different CTS induces distinct conformational changes in Na,K-ATPase (Klimanova et al., 2015), which may alter the nature of its interaction with partner proteins.

In contrast to CTS, which bind between the transmembrane helices of the Na,K-ATPase α-subunit, modeling data suggest that Aβ_42_ binds to the enzyme’s extracellular part, at the interface between its α- and β-subunits (Petrushanko et al., 2016; Adzhubei et al., 2022). The formation of a complex with ouabain does not affect the binding of Aβ_42_ to Na,K-ATPase (Adzhubei et al., 2022), which evidences that ouabain and Aβ_42_ have distinct binding sites on the enzyme and demonstrates their potential for simultaneous binding. Molecular modeling data demonstrated that the enzyme also binds Aβ_42_ when complexed with marinobufagenin, bufalin, or digoxin (Figure 4). While the interaction interface between Na,K-ATPase and Aβ_42_ is maintained upon CTS binding, the number of intermolecular contacts is changed (Figure 4). Among the considered CTS, digoxin exerts the most significant effect on the positioning of the Aβ_42_ bound to Na,K-ATPase. Conversely, the ouabain:Na,K-ATPase complex forms the smallest number of contacts with Aβ_42_ (Figure 4). A potential consequence of this is the ability of ouabain to prevent the Aβ_42_-induced activation of Src and the increase in APP levels. The greatest number of contacts between Na,K-ATPase and Aβ_42_ was observed in the presence of marinobufagenin and bufalin.

Marinobufagenin was the only investigated CTS that induced Src-activation and potentiated the effect of Aβ_42_ on APP levels (Figure 1). To investigate the reason for the opposing actions of ouabain and marinobufagenin on the Aβ_42_-induced increase in APP, the differences in their interaction with Na,K-ATPase should be considered, as well as the ways they influence the Aβ_42_ binding to the enzyme.

In the E2P conformation, Na,K-ATPase has a higher affinity for ouabain than for marinobufagenin. For instance, the dissociation constants for Na,K-ATPase (E2P) from duck salt glands are 0.1 μM for ouabain and 1.7 μM for marinobufagenin (Klimanova et al., 2015), while for Na,K-ATPase from pig kidneys, the values are 0.05 μM and 2.3 μM, respectively (Tverskoi et al., 2021). Marinobufagenin binds to different conformations of Na,K-ATPase with nearly equal affinity, including the E1 conformation, for which ouabain binding has not been detected. In contrast, ouabain exhibits high affinity specifically for E2P conformation of Na,K-ATPase (Klimanova et al., 2015). The lower sensitivity of marinobufagenin to the enzyme’s conformational state is associated with its shallower positioning within the binding site compared to ouabain (Klimanova et al., 2015; Tverskoi et al., 2021). This more superficial location of marinobufagenin in the binding site compared to all other investigated CTS was also confirmed for human Na,K-ATPase using molecular dynamics simulations (Figure 3). Furthermore, the binding of marinobufagenin to Na,K-ATPase, compared to ouabain, is characterized by a greater contribution of hydrophobic interactions to the binding energy and induces larger structural changes in the nucleotide-binding domain (Klimanova et al., 2015). Hence, ouabain and marinobufagenin induce distinct conformational changes in the Na,K-ATPase (Klimanova et al., 2015). Given that the Na,K-ATPase is known to interact with more than 10 protein partners (Reinhard et al., 2013), we hypothesize that these different conformations exhibit varying affinities for specific partners, such as PKC, BAX, and Bcl-2 affecting the enzyme activity and regulating its presence in the membrane (Lauf et al., 2015). This could explain the differences in the signaling and cytotoxic effects elicited by these CTS. For example, marinobufagenin induces cell death at significantly higher concentrations than ouabain, despite their similar half-maximal inhibitory concentrations (Akimova et al., 2005). Thus, the opposing effects of ouabain and marinobufagenin on Src activation and Aβ_42_-induced changes in APP levels are determined by differences in their affinity for various Na,K-ATPase conformations, as well as by conformational distinctions in the equilibrium states of Na,K-ATPase complexed with these ligands.

In murine models of AD, it the plasma level of marinobufagenin is decreased (Fedorova et al., 2020). Restoring this level via injections reduces neuroinflammation (Fedorova et al., 2020). Although there are currently no data on changes in endogenous CTS levels in the brains of AD patients, it is plausible that the decrease in plasma marinobufagenin in AD may result from impaired hippocampal function (Qi et al., 2024), as the hippocampus is a source of CTS in the brain.

Bufalin was the only investigated CTS that did not prevent the Aβ_42_-induced accumulation of APP in neurites (Figure 2). According to molecular modeling data, bufalin is positioned deeper within the Na,K-ATPase binding cavity than the other CTS (Figure 3), and, in its presence, Aβ_42_ forms the big number of contacts (about 293970) with Na,K-ATPase (Figure 4).

The potential use of cardiotonic steroids (CTS) in AD therapy has been considered previously, owing to their demonstrated ability to reduce neuroinflammation (for rev. see Poluektov et al., 2025), and their established, relative prevalence in the clinical management of other diseases, which is particularly widespread for ouabain and digoxin (Abaimov et al., 2024). Furthermore, the presence of ouabain and digoxin has been shown to reduce the production of tau protein, which is one of the key actors of AD development (Nguyen et al., 2023). Intraperitoneal administration of digoxin to rats with a sporadic model of AD protected them from memory impairments by preventing hippocampal cell death and reducing neuroinflammation and cholinergic deficiency (Erdogan et al., 2022). In addition, the ability of digoxin to eliminate senescent cells has been described, which may, in turn, contribute to neural repair in AD patients (Lee et al., 2023). On a molecular level, the affinity of digoxin for Na,K-ATPase is higher than that of marinobufagenin but lower than that of ouabain (Tverskoi et al., 2021). At the same time, digoxin, likewise ouabain, is a pseudo-irreversible inhibitor–in the contrast to marinobufagenin, which acts as a reversible inhibitor due to its more superficial binding (Tverskoi et al., 2021). It goes along well with our result that digoxin, the most extended of the investigated CTSs, is the most deepened in the Na,K-ATPase entry (Figure 3). Molecular modeling data also indicate that digoxin influences the subsequent binding of Aβ_42_ (Figure 4). Our data suggest that digoxin independently increases APP levels. Consequently, its long-term usage, even when administered against heart failure in Alzheimer’s patients, may accelerate pathologic accumulation of Aβ_42_ in the brain and consequently worsen the course of the disease.

Due to the redox-sensitivity of Src kinase (Heppner et al., 2018; Yang et al., 2020) and the ability of Na,K-ATPase glutathionylation to prevent its interaction with Src kinase (Petrushanko et al., 2017), the effect of CTS on ROS and GSH levels in Aβ_42_-treated cells was evaluated to see if the effects of some CTS are associated with altered redox status of the cells. However, all studied CTS slightly reduce GSH and ROS levels, exerting no effect on GSH and ROS levels while being combined with Aβ_42_. It means that the combined action of Aβ_42_ and CTS showed the same levels of GSH and ROS as those exposed to Aβ_42_ alone. Consequently, the effects of CTS on Src activation and APP level are not associated with changes in the redox status of cells.

The obtained data indicate that cardiotonic steroids (CTS) can directly influence APP levels and modulate the Aβ_42_-induced effects on its level. Whereas ouabain prevents the Aβ_42_-induced activation of Src kinase and the rise in APP level (Petrushanko et al., 2025), marinobufagenin, on contrary, potentiates the effect of Aβ_42_. Bufalin and digoxin, in their turn, do not activate Src kinase. These CTS lead to an elevation of APP by Src-independent way.

In all, our data indicate that CTS and Aβ_42_ modulate APP through distinct mechanisms. Bufalin and digoxin, in contrast to previously described Aβ_42_, elevate APP levels in a Src-independent way. Further, they are able to block Aβ_42_-induced Src kinase activation. On contrary, marinobufagenin itself activates Src kinase. This explains why marinobufagenin does not only block Aβ_42_-induced effects on APP levels but may even enhance it. Regarding APP subcellular localization, all tested CTS do not cause any significant alterations, in contrast to Aβ_42_, which robustly stimulates its accumulation in neurites. This effect can be resuppressed by ouabain, marinobufagenin, and digoxin. In conclusion, our findings demonstrate that endogenous CTS are involved in regulating APP levels and, consequently, the production of beta-amyloid in the brain.

## Data Availability

The datasets presented in this study can be found in online repositories. The names of the repository/repositories and accession number(s) can be found in the article/Supplementary Material.
