# Supplementary material for: Impact of the diverse cardiotonic steroids on beta-amyloid precursor protein level
Source: Front Pharmacol. 2025 Dec 11;16:1723629. doi: 10.3389/fphar.2025.1723629 (PMC12738930; doi:10.3389/fphar.2025.1723629)
Supplement: Supplementary file 1 [file Supplementaryfile1.docx]

Supplementary Material

# Supplementary Figures

**Supplementary Figure 1.** WST cell viability tests results after treatment SH-SY5Y neuroblastoma cells with digoxin (DGX), marinobufagenin (MBG) and bufalin (BUF) in the range of concentrations from 50 to 1000 nM. Mean values ± SD from at least three independent experiments are shown.

.


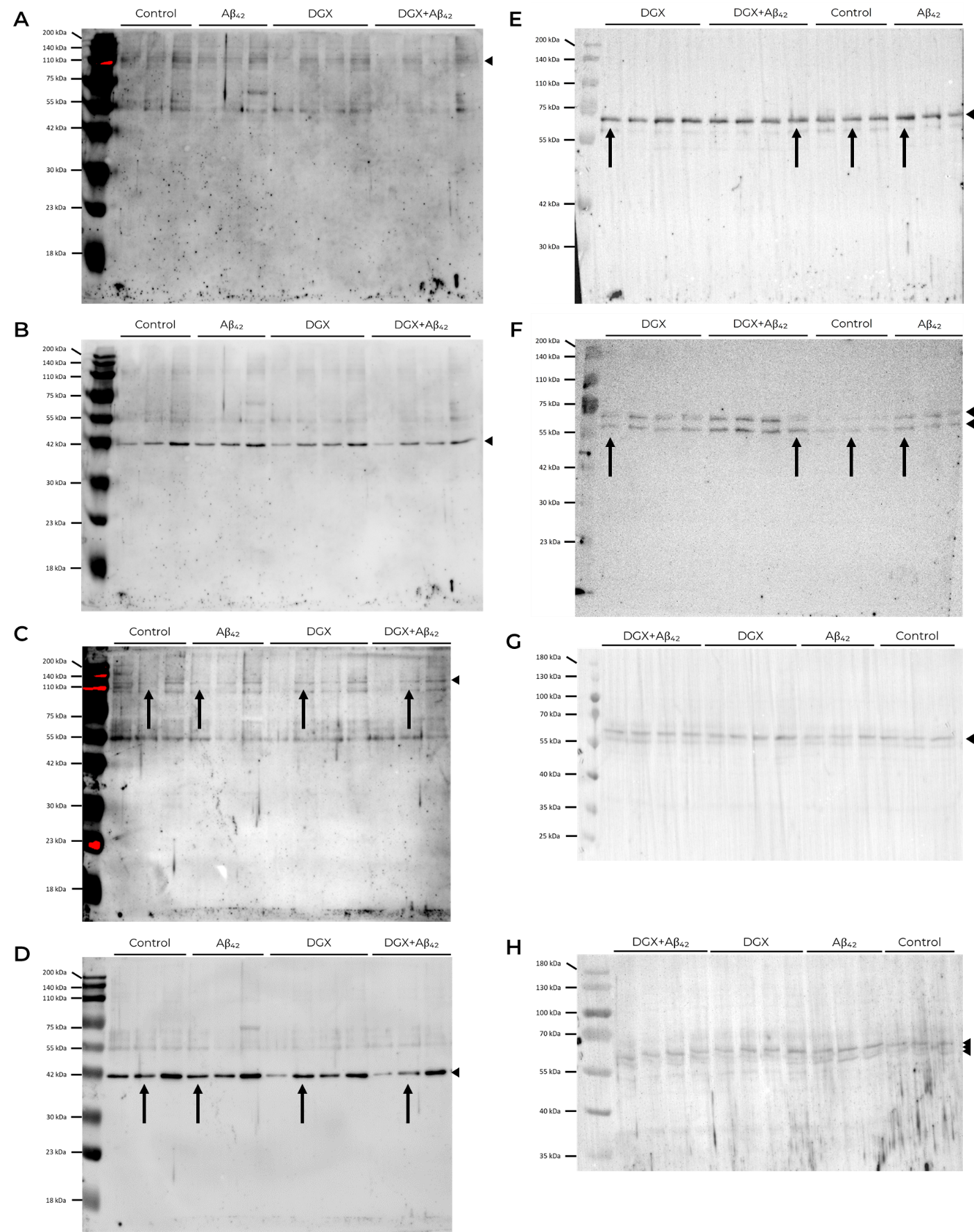


**Supplementary Figure 2.** Full-size Western-blot membranes (for Figure 1 A, B, G, H) with the control samples and the samples treated with 100 nM Aβ_42_, 100 nM digoxin (DGX) or both (DGX+Aβ_42_). Membranes were stained with the primary antibodies to APP (A, C), actin (B, D), Src kinase (E, G) and p(Tyr416)-Src kinase (F, H). The black triangles show the bands which were analyzed. Arrows show the representative bands which were shown in the Figure 1 A, G.


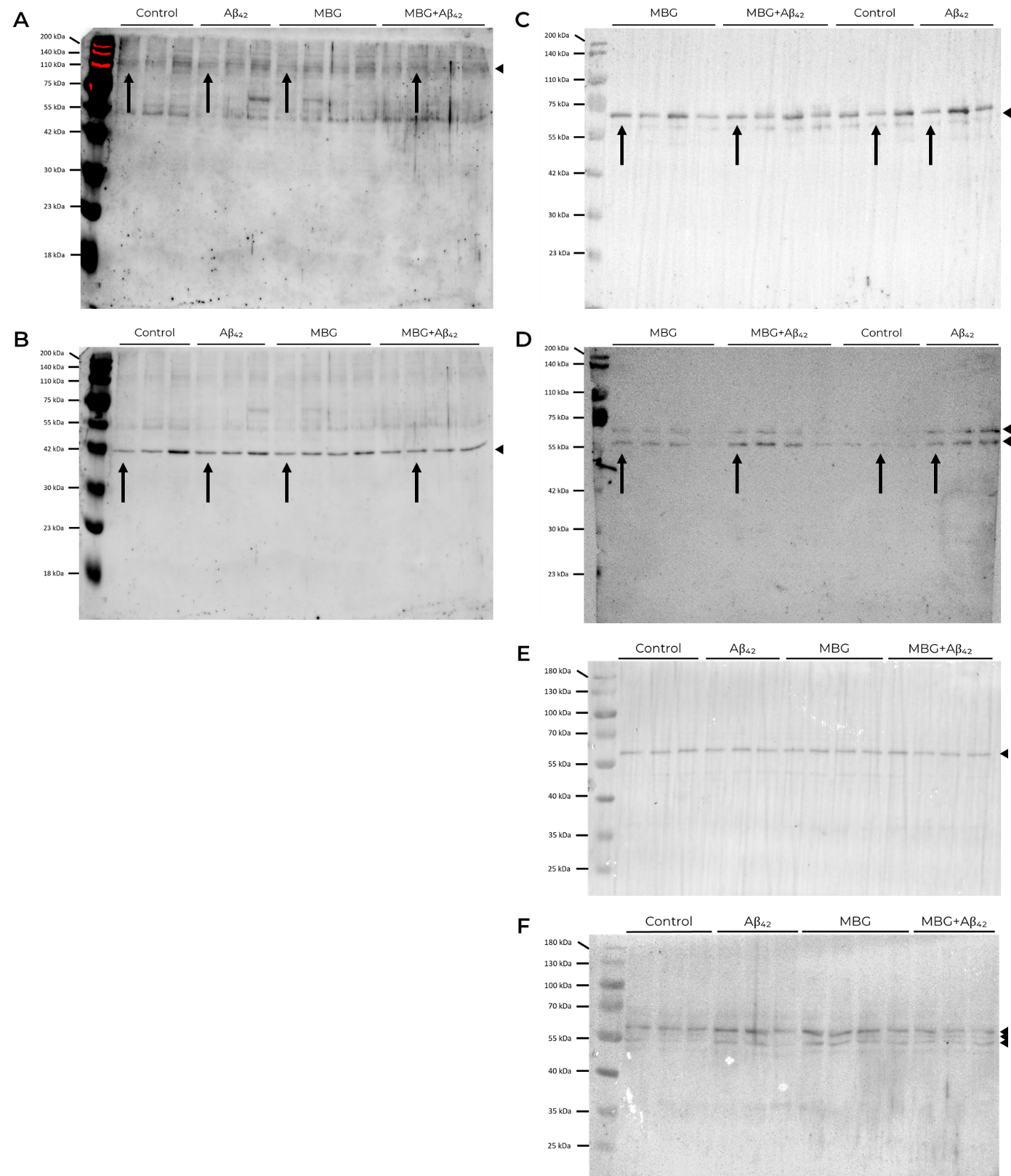


**Supplementary Figure 3.** Full-size Western-blot membranes (for Figure 1 C, D, I, J) with the control samples and the samples treated with 100 nM Aβ_42_, 100 nM marinobufagenin (MBG) or both (MBG+Aβ_42_). Membranes were stained with the primary antibodies to APP (A), actin (B), Src kinase (C, E) and p(Tyr416)-Src kinase (D, F). The black triangles show the bands which were analyzed. Arrows show the representative bends which were shown in the Figure 1 C, I.


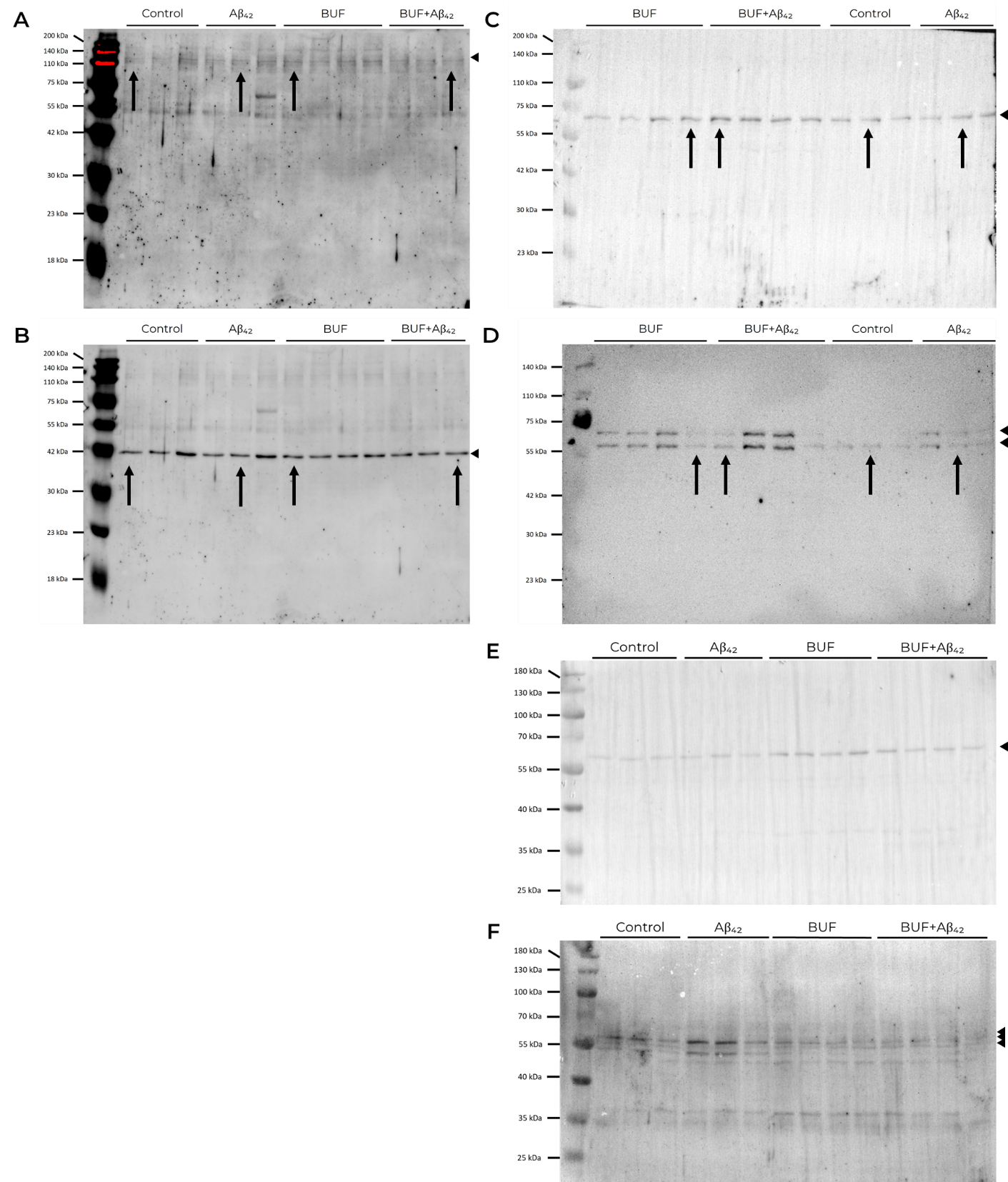


**Supplementary Figure 4.** Full-size Western-blot membranes (for Figure 1 E, F, K, L) with the control samples and the samples treated with 100 nM Aβ_42_, 100 nM bufalin (BUF) or both (BUF+Aβ_42_). Membranes were stained with the primary antibodies to APP (A), actin (B), Src kinase (C, E) and p(Tyr416)-Src kinase (D, F). The black triangles show the bands which were analyzed. Arrows show the representative bands which were shown in the Figure 1 E, K.


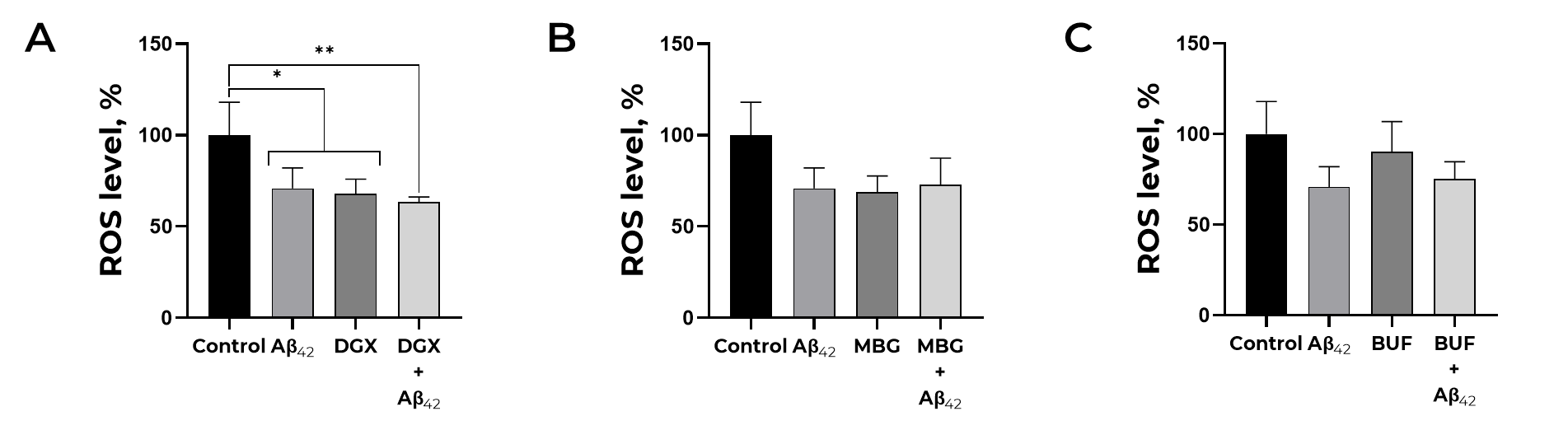


**Supplementary Figure 5.** Evaluation of the effect of Aβ₄₂ and cardiotonic steroids on the level of reactive oxygen species. (A) ROS levels in a presence of Aβ₄₂, digoxin (DGX) and their combination. (B) ROS level under amyloid and marinobufagenin (MBG) treatment. (C) Alterations in the ROS levels under exposure with Aβ_42_ and bufalin (BUF). The SH-SY5Y human neuroblastoma cells were harvested stained with dyhydrorhodamine 123 for ROS level measurements and incubated with 100 nM Aβ_42_ and, if required, 100 nM CTS for 30 minutes. All parameters were normalized for control. Mean values ± SD from at least three independent experiments are shown. * —p < 0.05, **—p < 0.01 compared to the control.


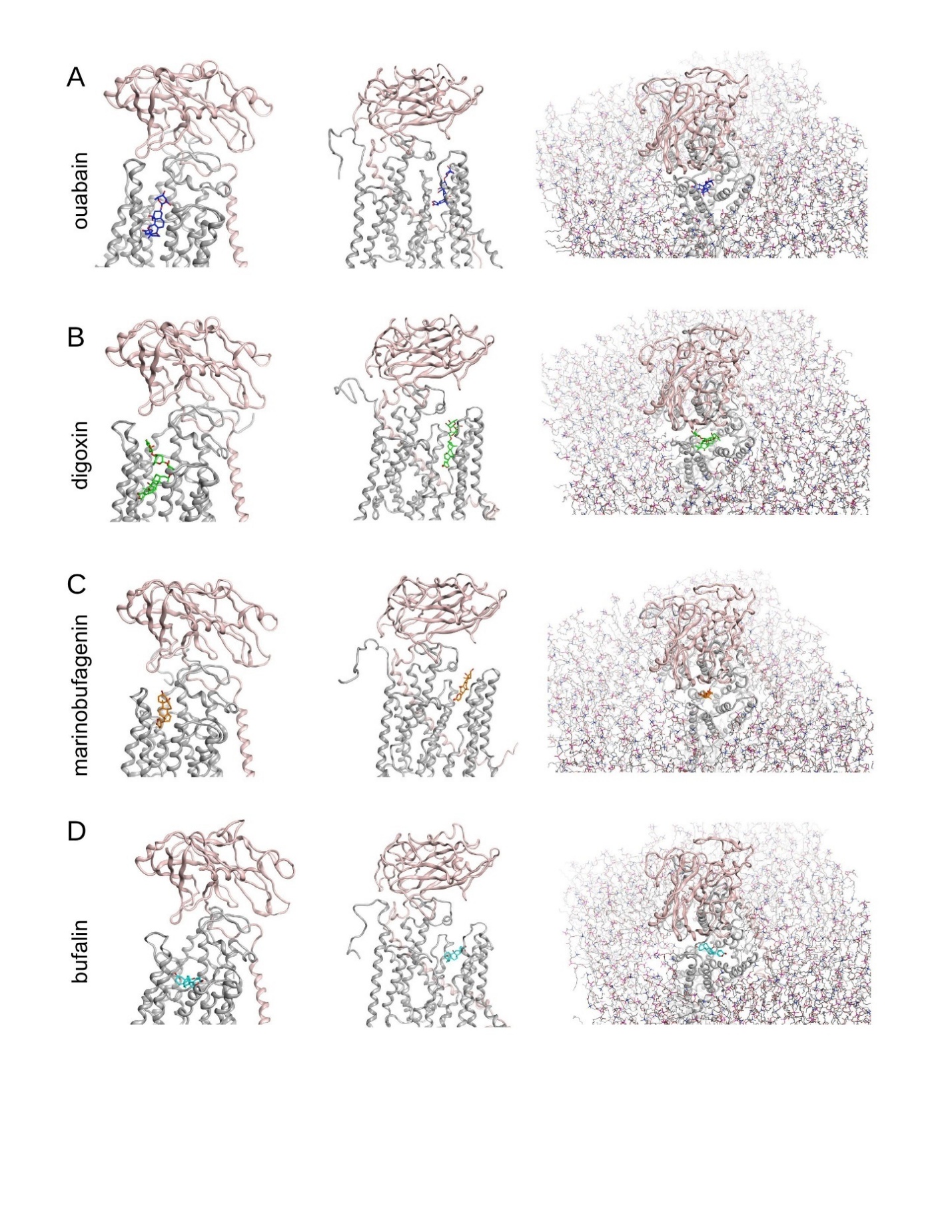


**Supplementary Figure 6**. CTS orientation in E2P Na,K-ATPase channel according to MD data. The centroid structures of Na,K-ATPase:CTS complexes were extracted from 100 ns MD trajectories using clusterization. Three projections are shown for each complex. Ouabain (A) is shown in blue, digoxin (B) in green, marinobufagenin (C) in orange, bufalin (D) is shown in cyan. All structures are superposed over 350 residues of the CTS binding site and extracellular surface near the entry into the channel as the potential Aβ_42_-binding site.


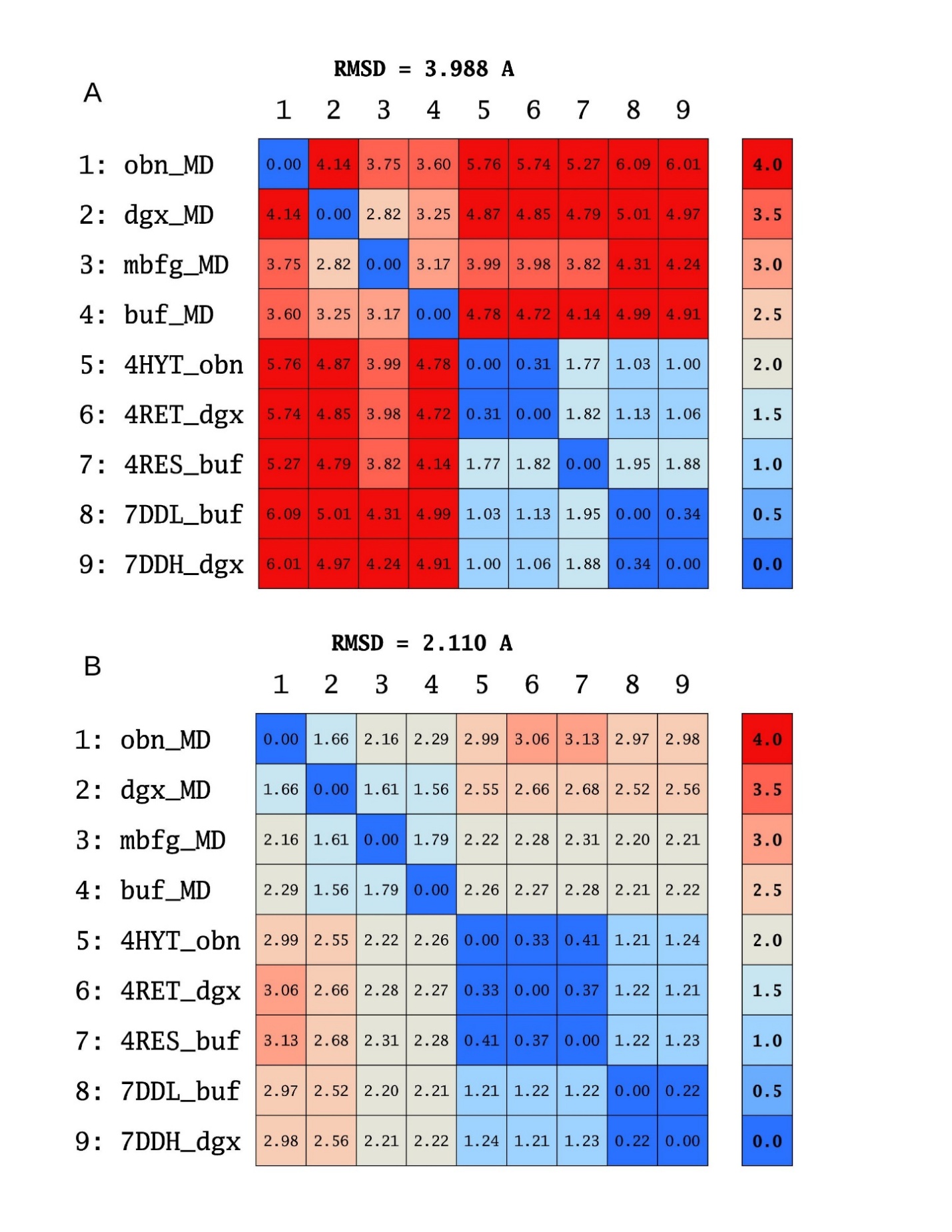


**Supplementary Figure 7.** Pairwise RMSD matrix between Na,K-ATPase structures in a complex with CTSs obtained by 100 ns MD (first 4 entries, CTS is marked on the table) and X-ray (4HYT PDB entry is for Na,K-ATPase:ouabain complex, 4RET PDB entry is for Na,K-ATPase:digoxin complex, 4RES PDB entry is for Na,K-ATPase:bufalin complex, 7DDL PDB entry is for Na,K-ATPase:bufalin complex, 7DDH PDB entry is for Na,K-ATPase:digoxin complex). (A) – RMSD calculated over 1317 residues for the whole structure presented in PDB bank. (B) RMSD calculated over 350 residues of the CTS binding site and extracellular surface near the entry into the channel as the potential Aβ_42_-binding site.

**Supplementary Table 1**. Interaction interfaces in Aβ_42_:Na,K-ATPase:CTS complexes obtained by docking. During docking, the position of the CTS was not considered, as docking servers do not handle engineered residues.

|  | OBN | DGX | MBFG | BUF |
| --- | --- | --- | --- | --- |
| Na,K-ATPase residues contacting with CTS | LEU132  LEU136  LEU318  ILE322  PHE323  VAL329  PHE790  LEU802  GLY803  THR806  ILE807 | ASN129  LEU132  VAL135  LEU136  ILE322  PHE323  ILE325  VAL329  PHE790  PHE793  LEU800  THR804  ILE807 | LEU318  ILE322 | LEU132  VAL329  PHE790  PHE793  LEU800  ILE807  LEU808 |
| Na,K-ATPase residue contacting with Aβ_42_ | LEU113  ILE117  THR316  TRP317  LEU318  ILE322  ARG887  VAL888  ASP889  ILE895  β-subunit:  GLN82  GLN84  PHE90  LYS111  LYS248 | ILE110  GLU111  TYR112  PHE113  ILE117  LYS120  GLU122  ASN129  PHE131  GLU314  THR316  ASP892  TRP894  ILE895 | ILE117  ILE312  LEU318  ARG887  VAL888  ASP889  ILE895  VAL898  GLU899  β-subunit:  ARG182  LYS248 | ILE117  GLU122  GLU314  ARG893  TRP894 |
| Aβ_42_ residues contacting with Na,K-ATPase | ASP7  TYR10  VAL12  HIS14  LYS16  LEU17  PHE19  GLU22  ASP23  LYS28  MET35 | ASP1  ALA2  ARG5  ASP7  TYR10  GLU11  VAL12  GLN15  LYS16  LEU17  VAL18  PHE19  PHE20  VAL24  ALA30  ILE31  ILE32  GLY37 | PHE4  ASP7  VAL12  LEU17  PHE19  ALA21  ASP23  LYS28  ALA30  ILE31 | ASP1  VAL18  PHE19  PHE20  GLU22 |
| Aβ_42_ residues intersect with CTS position | yes | yes | no | no |
